# Retroperitoneal fibrosis—the long and winding path

**DOI:** 10.1259/bjrcr.20190086

**Published:** 2020-09-29

**Authors:** Perawish Suwathep, Aazeb Khan, Rodwan Husein, Bella Huasen, Pentop Bose, Mark Brady

**Affiliations:** 1 Department of Radiology, Lancashire Teaching Hospitals, NHS Foundation Trust, United Kingdom; 2 Department of Vascular Surgery, Lancashire Teaching Hospitals, NHS Foundation Trust, City, United Kingdom; 3 Department of Renal Medicine, Lancashire Teaching Hospitals, NHS Foundation Trust, United Kingdom

## Abstract

Retroperitoneal fibrosis (RPF) is a rare systemic disease. Two-third of the cases are idiopathic but assumed to have autoimmune process related to IgG-4. It is often a diagnosis of exclusion due to its non-specific clinical presentation. Early manifestation commonly causes back pain, raised erythrocyte sedimentation rate level and renal impairment. Investigations of choice are MRI and contrast-enhanced CT but biopsy should be performed for diagnostic confirmation. This case report describes a delay in diagnosing RPF in a 57-year-old female who initially presented to primary care with back pain, mild anaemia, raised erythrocyte sedimentation rate and progressive renal function decline. She was seen urgently in haematology clinic who arranged bone scan to rule out osteoblastic metastases, finding demonstrated possible pelviureteric junction dysfunction. The investigation was followed by a MAG3 renogram 4 weeks later instead of an abdominal CT leading to diagnostic delay. She then presented acutely 1 day after renogram with life-threatening hyperkalaemia and AKI 3. RPF was then suspected. Renal ultrasound scan and CT scan consecutively showed bilateral gross hydronephrosis and retroperitoneal mass around the aorta. The pelviureteric junction dysfunction was due to ureters getting embedded into the dense retroperitoneal fibrous tissue. She subsequently underwent bilateral ureteric stent placement and was commenced on steroid therapy, with satisfactory outcome on follow-up. Laparoscopic retroperitoneal biopsy later confirmed the diagnosis. This case not only highlighted important learning points on the presenting features and radiographic findings of RPF, but also the clinician’s cognitive biases leading to diagnostic delay of a rare but life-threatening disease.

## Case report

### Clinical presentation

A 57-year-old Caucasian female patient presented to her General Practitioner (GP) with worsening lower back pain in the past 3 months. She was known to suffer from chronic mechanical back pain related to her job as a driver and also from a minor lumbar spine injury 15 years ago due to car accident. Initial blood investigations showed mild normocytic anaemia, with Haemoglobin (Hb) of 114 g l^−1^, and unexplained raised erythrocyte sedimentation rate (ESR) level of 71 mm/hr from her baseline of 33 mm/hr 3 months ago. This prompted her GP to refer her urgently to a Haematology team. Additionally, her renal function deteriorated from 6 months ago. Her creatinine increased from 52 to 88 mmol l^−1^ and estimated glomerular filtration rate (eGFR) decreased from 90 to 57 ml/min/1.73 m^2^. This was, however, not mentioned in the GP referral at the time.

She was seen at the haematology clinic within 2 weeks. With the history of acute on chronic back pain and mild anaemia, the differential diagnosis included underlying osteoporotic wedge fracture, possible inflammatory rheumatological condition and haematological malignancy such as multiple myeloma. Examination showed no lymphadenopathy or hepatosplenomegaly. Her anaemia was thought to be reactive caused by inflammatory response. Bone scan was then arranged to investigate possible myeloma bone disease. At this point, she was deemed suitable to be discharged from clinic pending additional investigations looking for haematological malignancy such as serum light chains and beta-2 microglobulins, which were later found negative. She also had repeated blood test at the clinic that showed further renal function decline. Her creatinine was now 95 mmol l^−1^ and eFGR was 53 ml/min/1.73 m^2^. Again, this was not commented upon during the clinic.

### Investigations/imaging findings

**Figure 1.**
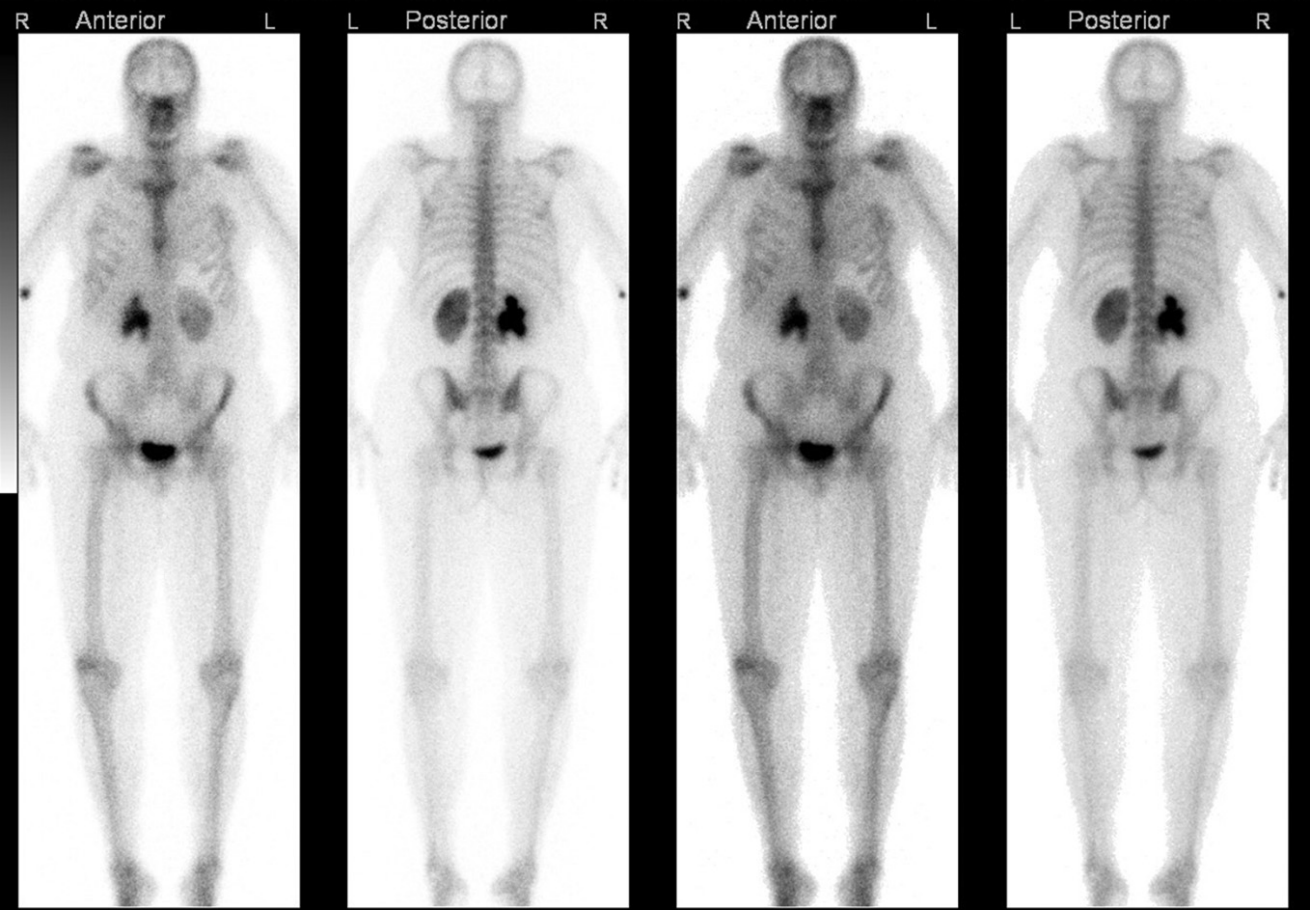
Nuclear medicine bone scan—no evidence of osteoblastic metastatic lesions. Retained tracer in the right pelvicalyceal system.

The bone scan happened 3 days post-haematology clinic which did not show evidence of osteoblastic metastases. Interestingly, retention of tracer in the right pelvicalyceal system was noted (Figure 1). The reporting radiologist described this as possible pelviureteric junction (PUJ) dysfunction and recommended mercaptoacetyltriglycine-3 (MAG-3) renogram as next investigation. The haematology team subsequently acted upon this advice and the renogram was arranged. In retrospect, these clinical courses lead to a delayed diagnosis that will later be discussed. Coincidentally, she also had pelvic ultrasound scan for post-menopausal bleeding in the following week which was unremarkable.

**Figure 2.**
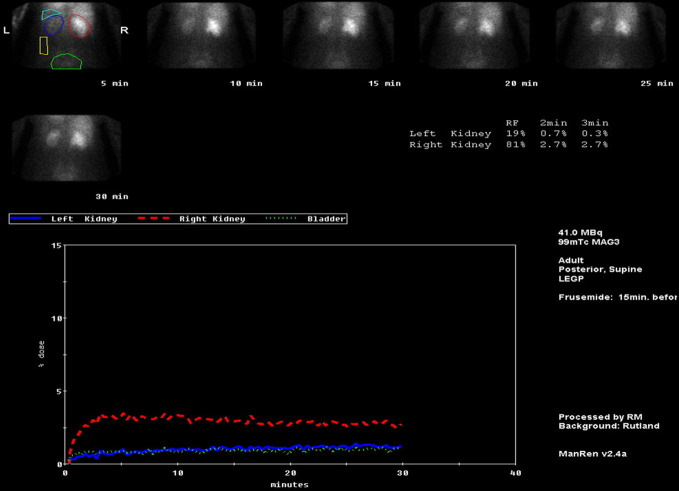
MAG3 renogram—red line demonstrates poor tracer uptake in the right kidney with minimal renal excretion. Blue line demonstrates significant reduced tracer uptake in the left kidney in comparison to the right kidney with minimal renal excretion. The static images show retained tracer in the right kidney at 30 min.

The MAG3 renogram was performed 4 weeks after the bone scan and the right kidney showed poor tracer uptake (which was less than 5%), with significant pooling of tracer in the collecting systems at 30 min and minimal excretion towards the end of the examination. There was a significant reduction of tracer uptake in the left kidney with corresponding low tracer excretion. The relative renal function for both kidneys were 81% on the right and 19% on the left (Figure 2). These features were in keeping with PUJ dysfunction with the left kidney much worse than right.

The differential diagnosis associated with PUJ dysfunction were secondary to pelvic mass or retroperitoneal mass effect, for instance from a lymphoma or metastatic disease. Infective cause such as local spread of tuberculosis from paraspinal abscess was considered. However, this was thought to be unlikely given the absence of infection signs and the demographics of this patient. Other less common differential diagnoses included multiple myeloma, sarcoma, carcinoid and histiocytosis.^1^

**Figure 3.**
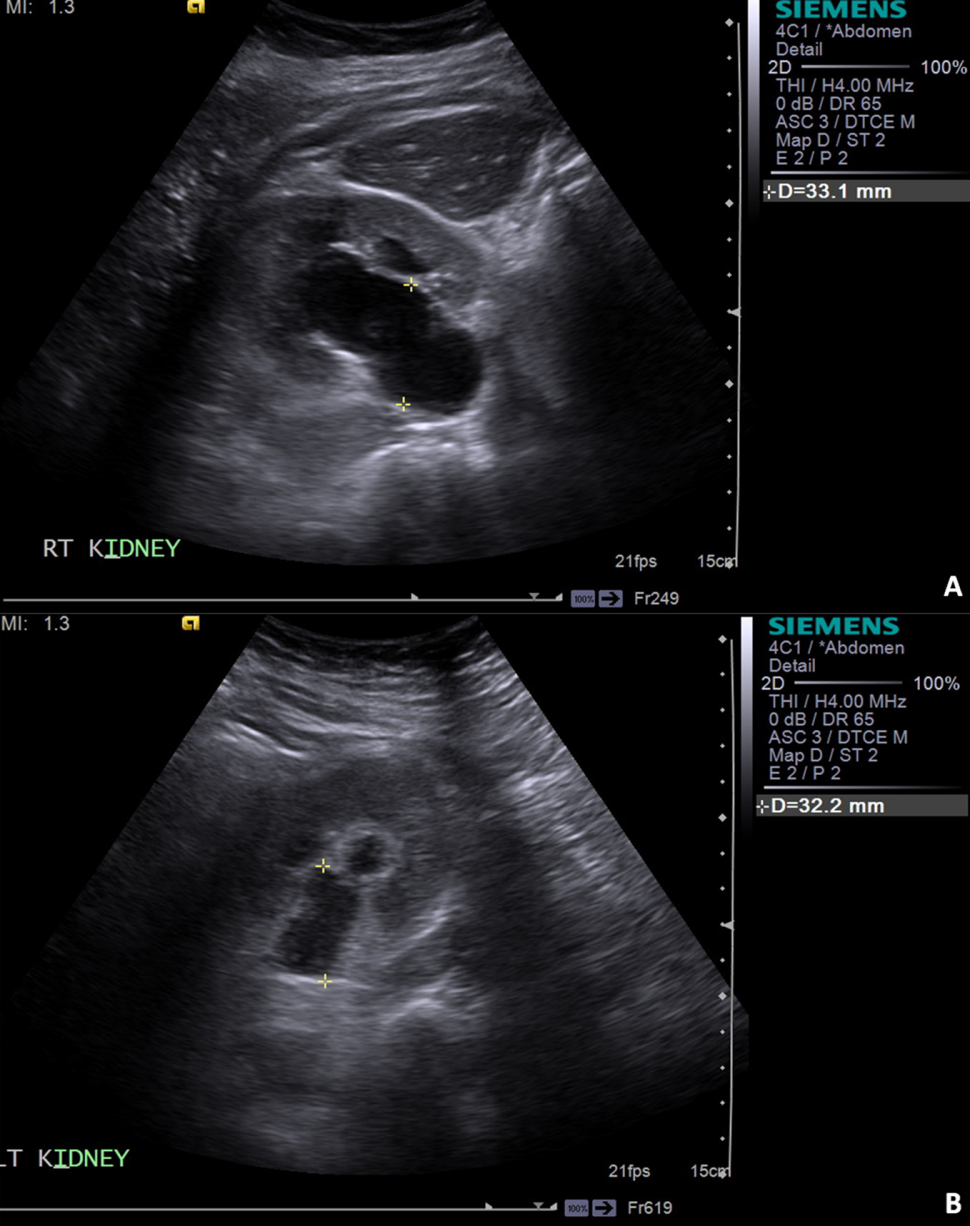
Ultrasound of renal tract. (**A**) Right renal pelvis measuring 33 mm in AP diameter. (**B**) Left renal pelvis measuring 32 mm in AP diameter.

The patient presented the following day after the renogram to the hospital with a life-threatening scenario. She was acutely unwell with Stage 3 acute kidney injury (AKI) and severe hyperkalaemia; with potassium level of 7.9 mmol l^−1^ and creatinine 686 mmol l^−1^. The hyperkalaemia required urgent interhospital transfer to her local renal centre to facilitate emergency haemodialysis. The possibility of suspected retroperitoneal fibrosis (RPF) was now raised (raised ESR, back pain, unexplained AKI with negative urinalysis). She had renal ultrasound scan (USS) which demonstrated grossly hydronephrotic kidneys bilaterally (Figure 3).

**Figure 4.**
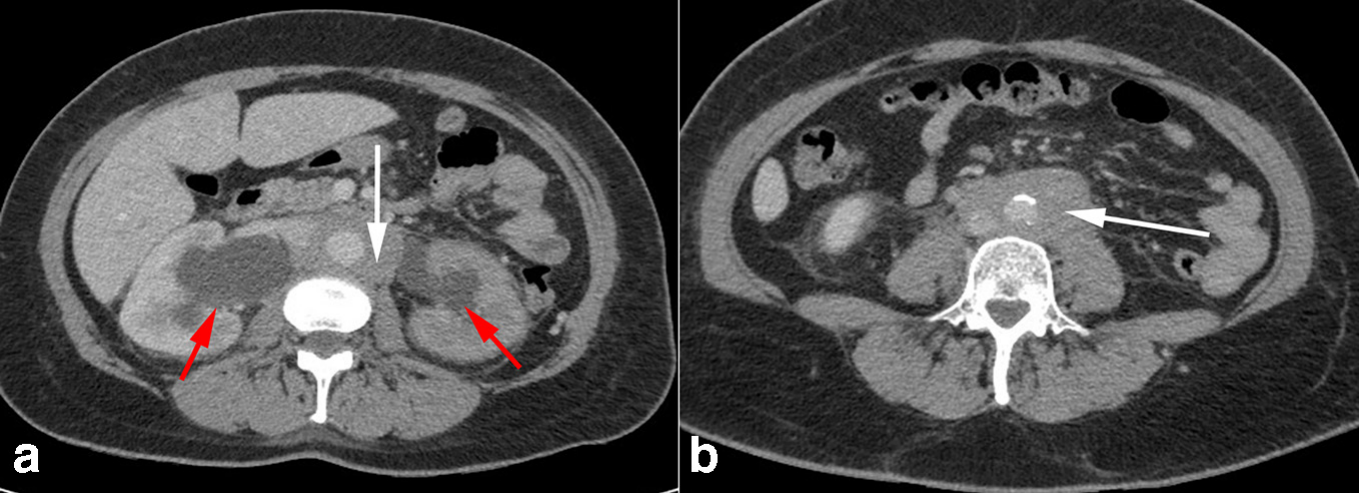
CT abdomen and pelvis portal venous phase. (a) Bilateral hydronephrosis (red arrows) with soft tissue density surrounding the aorta and inferior vena cava (white arrow). (b) Retroperitoneal soft tissue density extending into pelvis (arrow).

Dialysis was provided via temporary right internal jugular catheter after several unsuccessful right femoral cannulation attempts. Right groin USS performed to assess damage showed 27 × 17 × 29 mm pseudoaneurysm. Subsequent CT scan to assess hydronephrosis was modified to include CT angiogram to assess right femoral artery damage. Findings were reported as bilateral severe hydronephrosis with poor enhancement of the left kidney. A rind of soft tissue around the aorta, inferior vena cava and iliac vessels was noted without evidence of vascular invasion (Figure 4). The CT scan confirmed the classic appearance of a retroperitoneal mass around the aorta which did not enhance in the arterial phase.^2,3^ There was no significant para-aortic lymphadenopathy, hence unlikely to represent lymphoma.^4^

**Figure 5.**
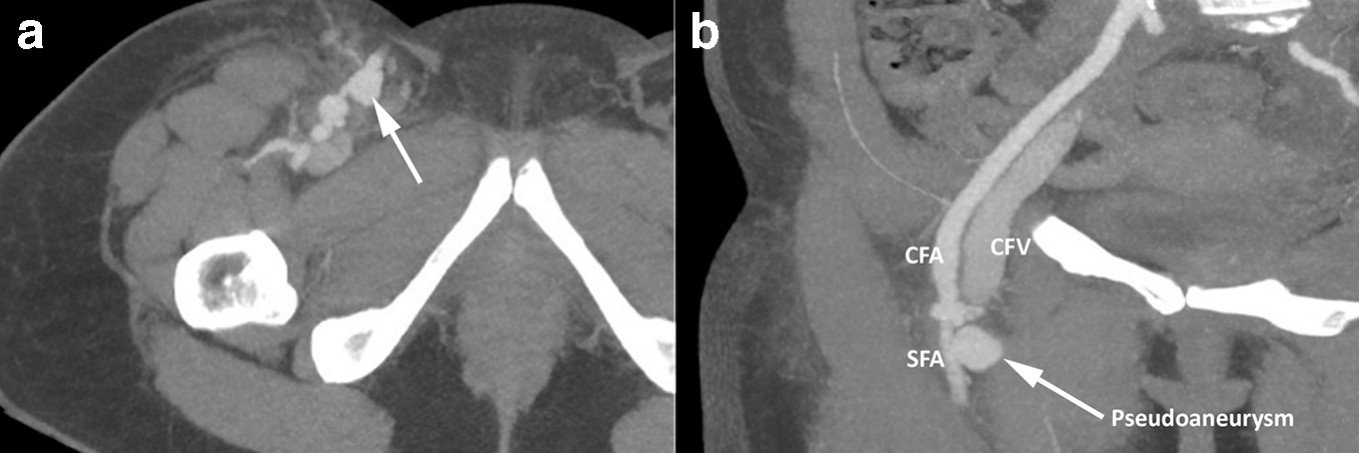
CT angiogram. (a) Enhancement of the right SFA, with abnormal saccular arterial density arising from the right SFA in keeping with SFA pseudoaneurysm (arrow). (b) Coronal image demonstrating right SFA pseudoaneurysm (arrow) and early enhancement of the right CFV. CFA, common femoral artery; CFV, common femoral vein; SFA, superficialfemoral artery.

The CT angiogram also demonstrated a lobulated pseudoaneurysm measuring a maximum of 4 cm craniocaudally in the right groin. This was arising from the anterior wall of the superficial femoral artery where the line insertion attempt had been made (Figure 5). Additionally, there was early venous filling of the right common femoral vein and right external iliac vein consistent with an arteriovenous fistula, although the actual site of connection was difficult to ascertain.

### Treatment

Bilateral ureteric stents were inserted 1 day post dialysis via a retrograde ureteropyelogram in order to relieve the PUJ obstruction caused by the soft tissue mass effect. Additionally, the right femoral artery open repair was also performed at the same time. Retroperitoneal fibrosis was treated with immunosuppression using oral prednisolone 40 mg once daily. The patient was discharged on Day 7 and her renal function significantly improved (creatinine 111 mmol l^−1^).

### Outcome and follow-up

The patient had an early and then regular outpatient reviews post-discharge. There was a continuous improvement in renal function, with creatinine of 74 mmol l^−1^, eGFR of 70 ml/min/1.73 m^2^ and ESR 12 mm/hr at 3 months post-discharge.

**Figure 6.**
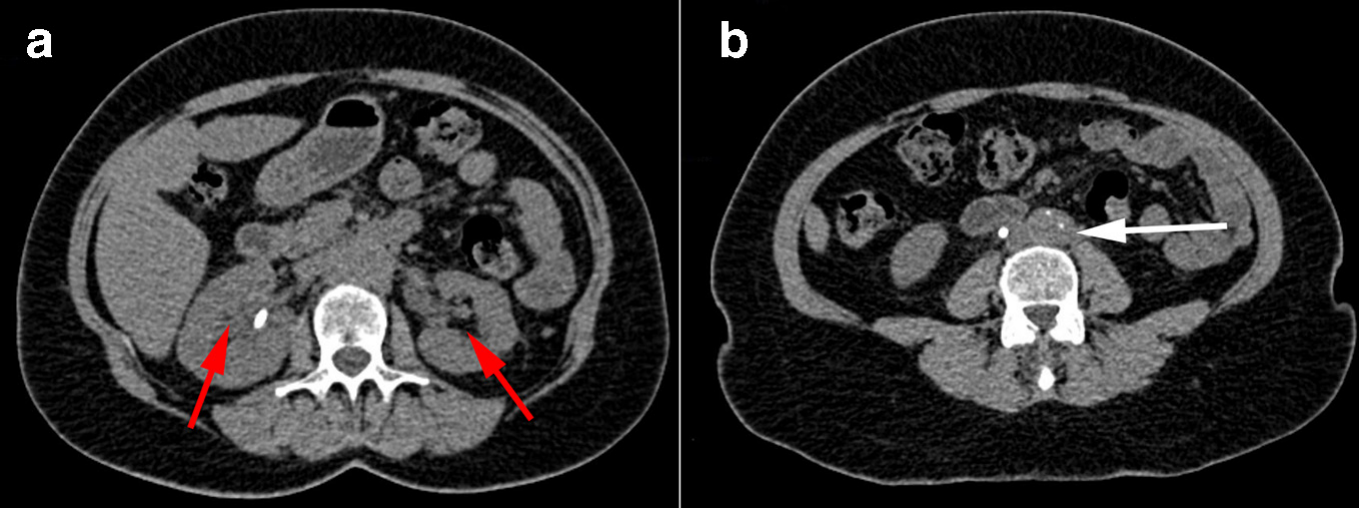
Follow-up CT KUB 1 year after treatment. (a) Resolution of hydronephrosis (red arrows). (b) Significant reduction of retroperitoneal soft tissue density (arrow) compared to Figure 4B at the same level. KUB, kidneys, ureters and bladder.

Follow-up CT guided biopsy for RPF at 1 month was cancelled due to not having safe window for access and converted to laparoscopic retroperitoneal biopsy 2 months after. At the same time, she received left ureterolysis with omental wrap exposing the proximal ureter from retroperitoneum to relieve its pressure. The histology sample confirmed prominent collagen bundles and chronic inflammatory cells, mostly lymphocytes, in keeping with retroperitoneal fibrosis.^4^ She had follow-up CT KUB (Kidneys, Ureters and Bladder) 1 year after treatment showing significant reduction of RPF and resolution of hydronephrosis (Figure 6). She continued to have 4–6 monthly ureteric stents replacement and they were eventually removed 2 years later.

### Discussion

Retroperitoneal fibrosis (Ormond's disease) is a disease of unknown aetiology but assumed to be an autoimmune process related to IgG-4 with periaortitis as the initial pathophysiological mechanism.^1,3^ It has been linked to many causes such as certain medications, infections, inflammatory disorders especially in advanced atherosclerotic disease and malignancies.^4^

The literature describes patients often presenting with non-specific symptoms such as back or abdominal pain, and then renal failure at a later stage. ESR levels tend to be elevated. The non-specific clinical manifestations pose difficulties in early diagnosis.^3,5^ Such is its rarity that, naturally, more common causes must be excluded before making the diagnosis. The presenting symptoms of this patient were also compatible with her pre-existing co-morbidity of chronic back pain and post-menopausal bleeding (which may cause anaemia), contributing to further diagnostic challenge.

Upon analysing the clinical courses, this patient would have clearly benefited from early abdominal evaluation such as CT abdomen or abdominal ultrasound after the initial bone scan result. Particularly, in the context of suspected haematological malignancy and abnormal renal function. This case report reflects upon the several cognitive biases leading to diagnostic delay. In this instance, the GP referred her to haematologist for back pain, high ESR and anaemia without mentioning the renal function, resulting in "Framing bias". This is a cognitive effect when clinicians come to decision based on how the information is initially presented and may account for why the renal function decline were not addressed at the time.^6^ As haematology is a super-specialised field, the patient was initially investigated by bone scan primarily from haematological perspective (such as myeloma), which is an example of "availability bias". This is a common occurrence when clinician favours a course of action based on readily available knowledge and familiarity.^7,8^ Moreover, the reporting radiologist recommended MAG3 renogram to further assess the suspected PUJ dysfunction which was not the most appropriate diagnostic scan in context of suspecting malignancy and progressive renal function decline. This was likely due to "anchoring bias" when clinicians focused exclusively on a single finding.^8^ This is when "diagnostic momentum" occured (continuing the radiologist’s recommendation without re-evaluation) leading to diagnostic delay.^6^ As a result, other differential diagnosis causing renal function decline were not fully assessed. These cognitive effects are an important learning point particularly in the world of increasing super-specialisation. Each step was valid in isolation, but like a swiss cheese model in this case aligned towards a life-threatening presentation.^9^

There are currently no set guidelines on how to approach the diagnosis of RPF. Most of the current literature identifies CT and MRI as the most useful tools in confirming RPF.^2,10^ Previously, fluoroscopy was used to perform intravenous urogram. Findings of hydronephrosis or even delayed contrast excretion into the collecting system and medial deviation of the middle ureters can be appreciated along with tapering of ureteric lumen around L4-S1.^6^ Now CT imaging is the main modality for investigating RPF. On portal-venous phase imaging a retroperitoneal soft tissue density homogeneous mass is noted, typically encasing the infra renal abdominal aorta and iliac vessels moving cranially towards the renal hila. This soft tissue changes do not invade these vascular structures or the ureters.^2,10^ Other diseases which can present with similar radiological findings include retroperitoneal lymphoma, which is typically centred more cranially to L4-5, and rarely retroperitoneal extramedullary haematopoiesis, seen as a retroperitoneal mass but displaces the ureters laterally and the aorta anteriorly from the vertebrae spine.^10^

MRI is also useful though not as easily accessible, has the added advantage of better contrast resolution. The sensitivity is equal to that of CT imaging.^10^ RFP appears dark, low intensity on *T*_1_ and *T_2_* weighted images, and in the presence of active inflammatory process the *T_2_* weighted signal can be variable or hyperintense.^2,11^

In active inflammatory phase, where metabolic activity is high, RPF can show avid radiotracer fludeoxyglucose uptake in positron emission tomography (PET)-CT imaging. Though not a specific nor sensitive test for RPF, it has the advantage of demonstrating active disease and follow up assessment.^10^

As the disease progresses, the fibroinflammatory mass extends into the retroperitoneum, enveloping the adjacent structures, with the ureters being the most affected, causing associated proximal hydroureter and hydronephrosis.^12^ Displacement of the proximal and mid-ureter to the mid-line on imaging is typical of RPF and should trigger a differential of this disease.

The soft tissue mass may demonstrate varying degree of enhancement in CT depending on the stage of the disease. During the acute stage, there is avid enhancement of the soft tissue mass with little or no enhancement seen in the later or chronic stage.^2^ However, patients with RPF often presents with poor renal function due to obstructive uropathy, which causes the debate whether i.v. contrast agents should be administered.^5^ In case of non-specific imaging features, a biopsy is indicated, which remains the mainstay for diagnostic confirmation, allowing a confident differentiation of benign or malignant RPF.^1,2^

The initial acute management of RPF is decompression of hydronephrosis with insertion of “double J” ureteric stents. The mainstay of medical treatment is by using immunosuppressants to inhibit progression of the fibroinflammatory reaction caused by RPF.^1^ Corticosteroids are commonly used and has been shown to improve the obstructive symptoms and reduce the size of retroperitoneal mass.^1,12^ Other immunosuppressants such as azathioprine and cyclophosphamide can be used in recurrent disease or when corticosteroids are contraindicated. Surgical management to relieve ureteral obstruction is indicated in a patient who failed to improve with medical treatment. This is usually done by open ureterolysis with intraperitoneal transposition and follow by omental wrapping of the ureters.^1^ Surgical approach also allows multiple retroperitoneal biopsies to be carried out if diagnosis remains unclear.

Overall, retroperitoneal fibrosis is uncommon and carries good prognosis following appropriate treatment when there is no underlying malignant cause. It should be included in a broad differential diagnosis for a patient with unexplained impaired renal function and ureteric deviation or PUJ dysfunction. Currently, there are no set pathways or guidelines available to aid clinicians in the diagnosis of RPF. Further study is needed for a better understanding of this disease. Cognitive biases can occur in multispecialty settings leading to diagnostic delay. The current practice of increasing subspecialisation and specialty referrals may once more lead to a long and winding path to diagnosis without vigilance and taking an overview.

## Learning points

1. Retroperitoneal fibrosis is a rare disease often presents with non-specific symptoms causing diagnostic difficulty. It should be considered in a patient presenting with back pain, raised ESR and renal impairment. Progressive deterioration in creatinine and eGFR without a clear cause may reflect its early sign that should prompt urgent nephrology and urology referral.
2. CT imaging is the main modality for investigating retroperitoneal fibrosis. Findings often consist of a retroperitoneal soft tissue density mass encasing the infrarenal abdominal aorta moving cranially towards the renal hila.
3. Retroperitoneal fibrosis typically causes PUJ dysfunction and medial deviation of the ureter without aortic displacement and usually centred around L4-5 region.
4. Cognitive biases such as framing, availability, anchoring and diagnostic momentum could explain clinicians’ behaviour leading to diagnostic delay. This is particularly common in current practice where multiple specialty referrals are involved.
